# Intertrochanteric fractures treated with proximal femoral bionic nail versus proximal femoral nail antirotation versus InterTan Nail: A meta-analysis

**DOI:** 10.1016/j.jor.2026.03.020

**Published:** 2026-03-19

**Authors:** Thomas Cho, Kriti Devgan, Nipun U. Jayatissa, David Yatsonsky, Jiayong Liu

**Affiliations:** Department of Orthopedic Surgery, University of Toledo Medical Center, Toledo, OH, 43614, USA

**Keywords:** Intertrochanteric fracture, Proximal femoral bionic nail, Proximal femoral nail antirotation, InterTan, Outcomes

## Abstract

Intertrochanteric fractures, common in the elderly, are often treated with intramedullary (IM) nails. The comparison of outcomes for different IM nails has not been thoroughly evaluated. This meta-analysis compares postoperative outcomes of PFNA, InterTan, and the newer PFBN. PubMed, Embase, and Google Scholar were searched until April 2025. Comparative studies evaluating ≥2 IM nails reporting key outcomes were included. Outcomes included union/weight-bearing/hospital stay times, operative time, blood loss, complications (delayed/non-union, hardware failure, infections, deep vein thrombosis, femoral head necrosis, screw cutout), and Harris Hip Score (HHS) at 6/12 months. Analyses used Review Manager 5.4: mean difference (MD) for continuous variables (inverse variance) and risk ratio (RR) for dichotomous variables (Mantel-Haenszel). Significance: *P* ≤ 0.05. Twenty-six studies, involving 2690 patients, were analyzed. When comparing PFBN to PFNA, PFBN had shorter union (*P* = 0.01) and weight-bearing times (*P* = 0.003), but longer operative time (*P* = 0.003). When comparing PFBN to InterTan**,** PFBN showed superiority in union (*P* < 0.001), weight-bearing (*P* < 0.001), hospital stay (*P* < 0.001), operative time (*P* < 0.001), blood loss (*P* < 0.001), and HHS at 6 (*P* = 0.003) and 12 months (*P* = 0.009). When comparing PFNA to InterTan, InterTan had shorter union (*P* = 0.001) and weight-bearing (*P* = 0.03) times, fewer screw cutouts (*P* < 0.001); PFNA had shorter operative time (*P* = 0.002) and less blood loss (*P* = 0.004). Secondary analysis of unstable fractures yielded similar results. PFBN demonstrates superior postoperative outcomes compared to PFNA and InterTan. Further studies comparing PFBN with other IM nails are needed to establish optimal clinical use.

**Level of evidence:**

3

## Key points

• **Objective:** This meta-analysis compared outcomes of three IM nails (PFNA, InterTan, and PFBN) for elderly intertrochanteric fractures, using data from 26 studies (2690 patients).
• **PFBN vs. PFNA:** PFBN offered faster recovery (union and weight-bearing) but required a longer operative time.
• **PFBN vs. InterTan:** PFBN was superior across almost all metrics, including faster recovery, shorter surgery, less blood loss, and better functional scores.
• **PFNA vs. InterTan:** Results were mixed; InterTan enabled faster healing with fewer screw cutouts, while PFNA had shorter operative times and less blood loss.
• **Conclusion:** The newer PFBN showed superior postoperative outcomes overall, though more research is recommended to confirm its optimal clinical use.

## Graphical abstract

Meta-analysis of 2690 patients shows the Proximal Femoral Bionic Nail (PFBN) outperforms Proximal Femoral Nail Antirotation (PFNA) and InterTan.PFBN vs. Alternatives:• Faster fracture union & weight-bearing.• Better functional hip scores.• Superior in unstable fractures.PFBN demonstrates the most favorable outcomes for intertrochanteric fracture fixation.

**Figure:**
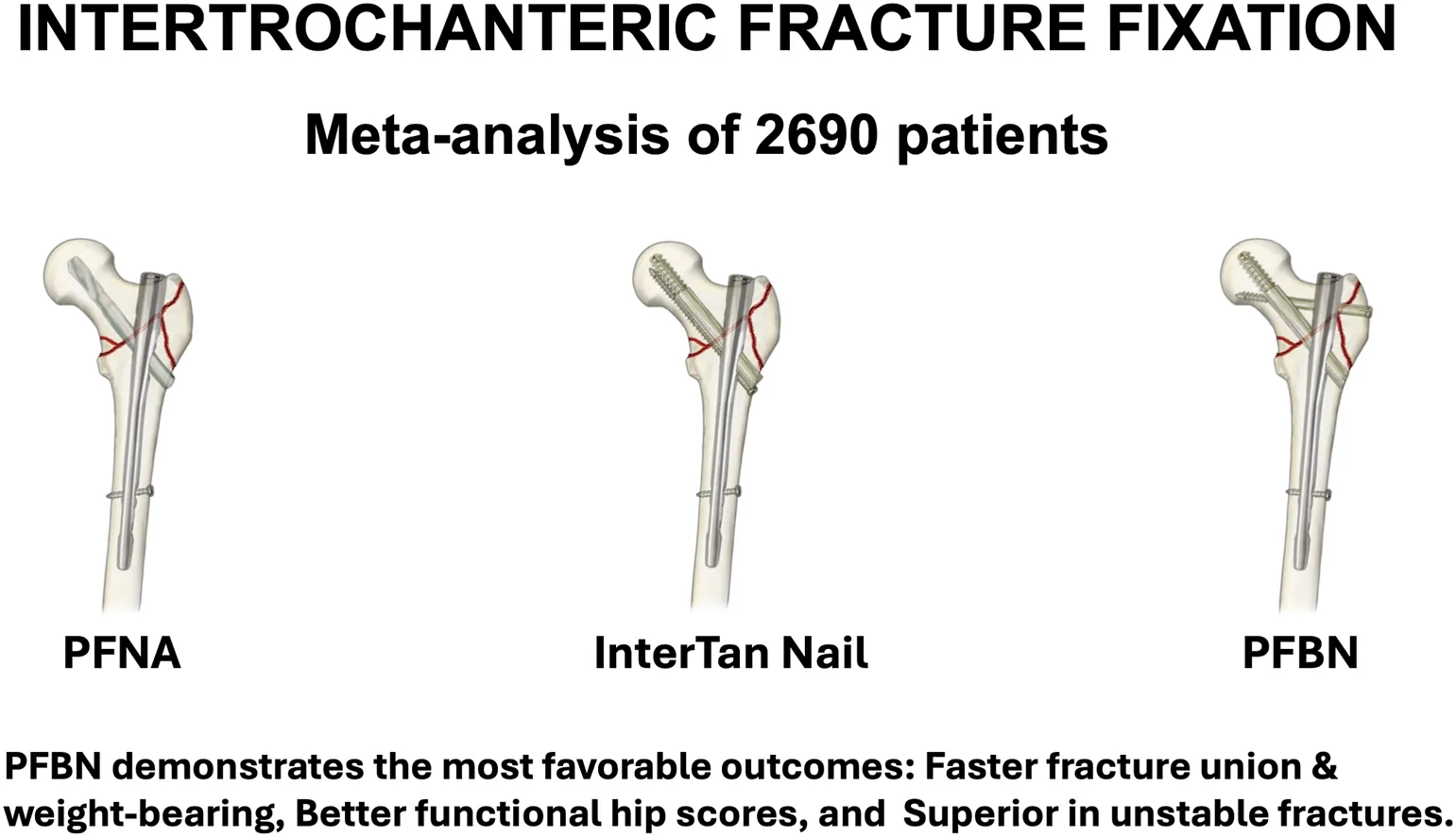

## 1 Introduction

Hip fractures are common injuries seen in the elderly, with the number of fractures likely to increase due to the aging global population.^1^^,^^2^ Hip fractures are divided into two main types, intracapsular and extracapsular, where the former occurs within the hip joint capsule and the latter occurs outside. Intertrochanteric fractures (IFF) are a type of extracapsular fracture that specifically occur between the greater and lesser trochanters of the femur, with it making up approximately 50% of all hip fractures.^3^^,^^4^ The two main surgical interventions for IFF include extramedullary and intramedullary fixation, and its indications depend on the stability. A stable IFF (AO/OTA 31-A1) is characterized by a simple fracture pattern with no significant displacement or comminution, and extramedullary fixation has been shown to have similar functional outcomes but better cost-effectiveness, while unstable IFF (AO/OTA 31-A2 and 31-A3) are more complex with patterns such as reverse-oblique orientation and large comminution, where intramedullary fixation has shown to have better outcomes.^5^^,^^6^

**Fig. 1:**
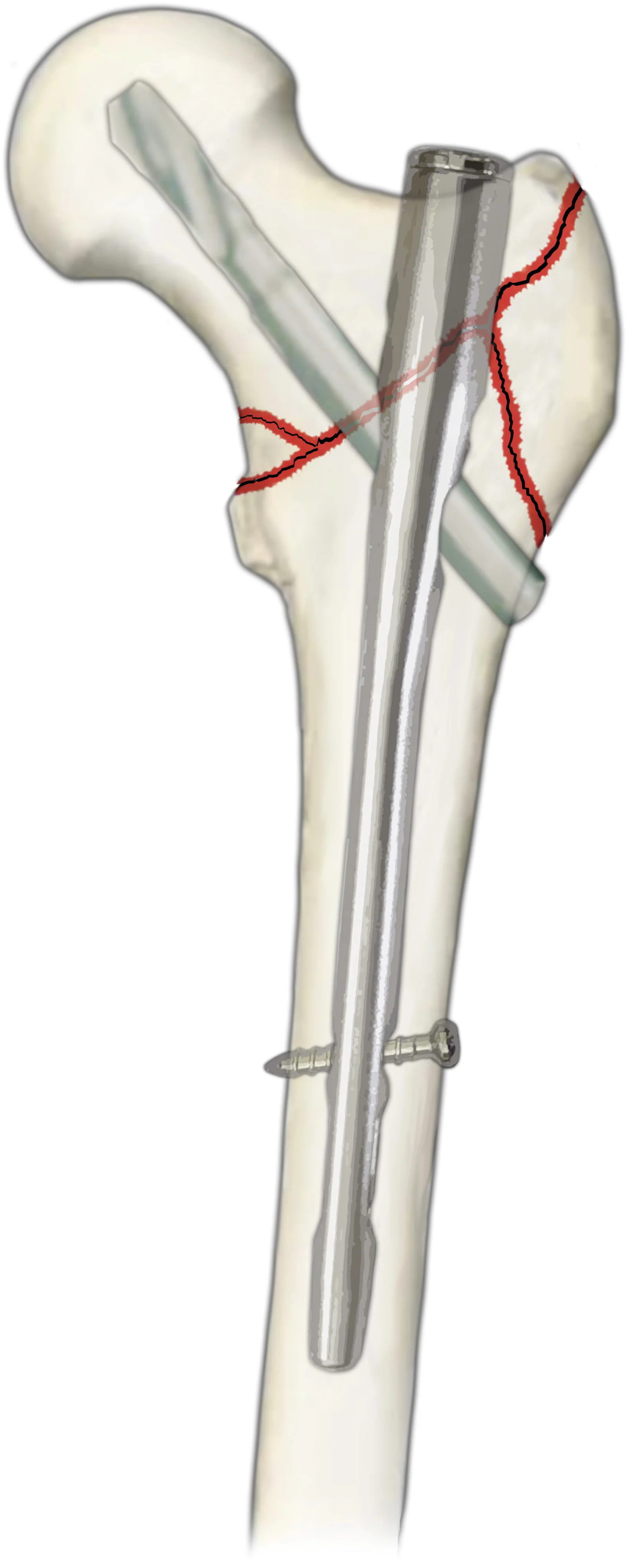
Illustration of Proximal Femoral Nail Antirotation (PFNA).

**Fig. 2:**
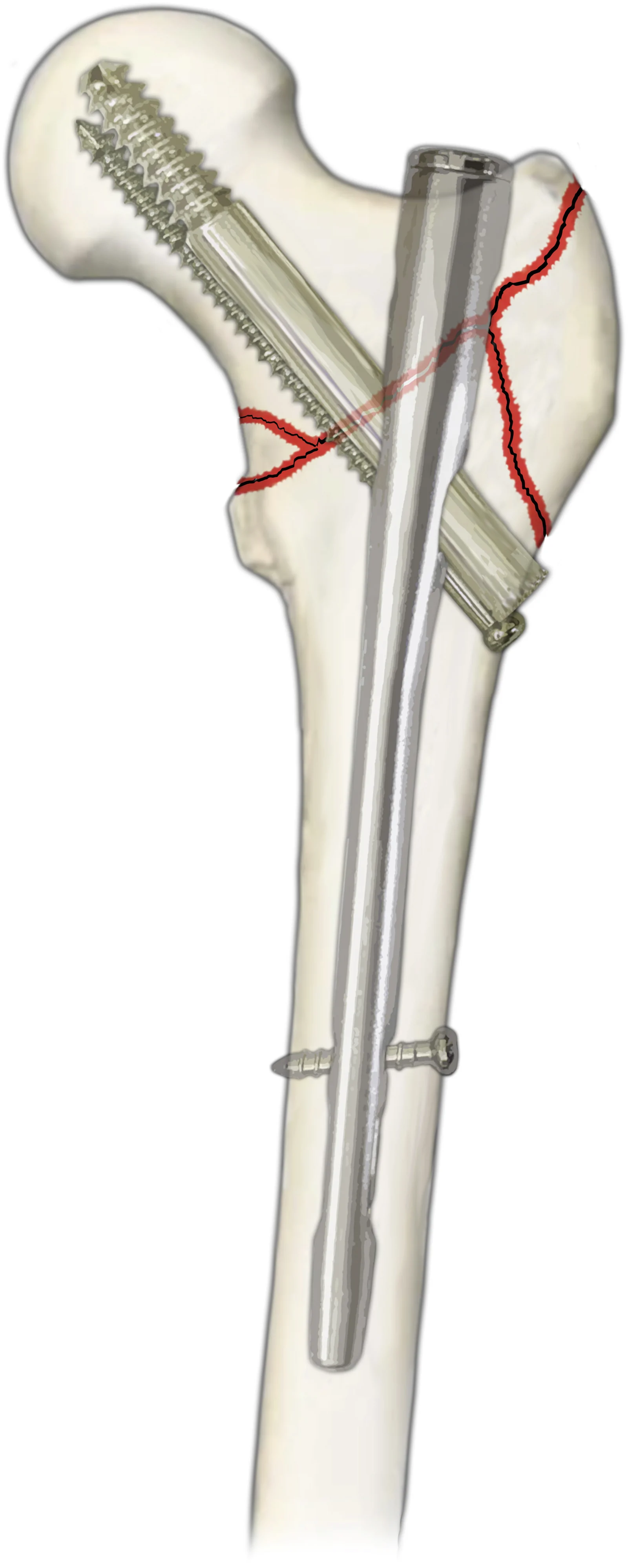
Illustration of InterTan Nail.

**Fig. 3:**
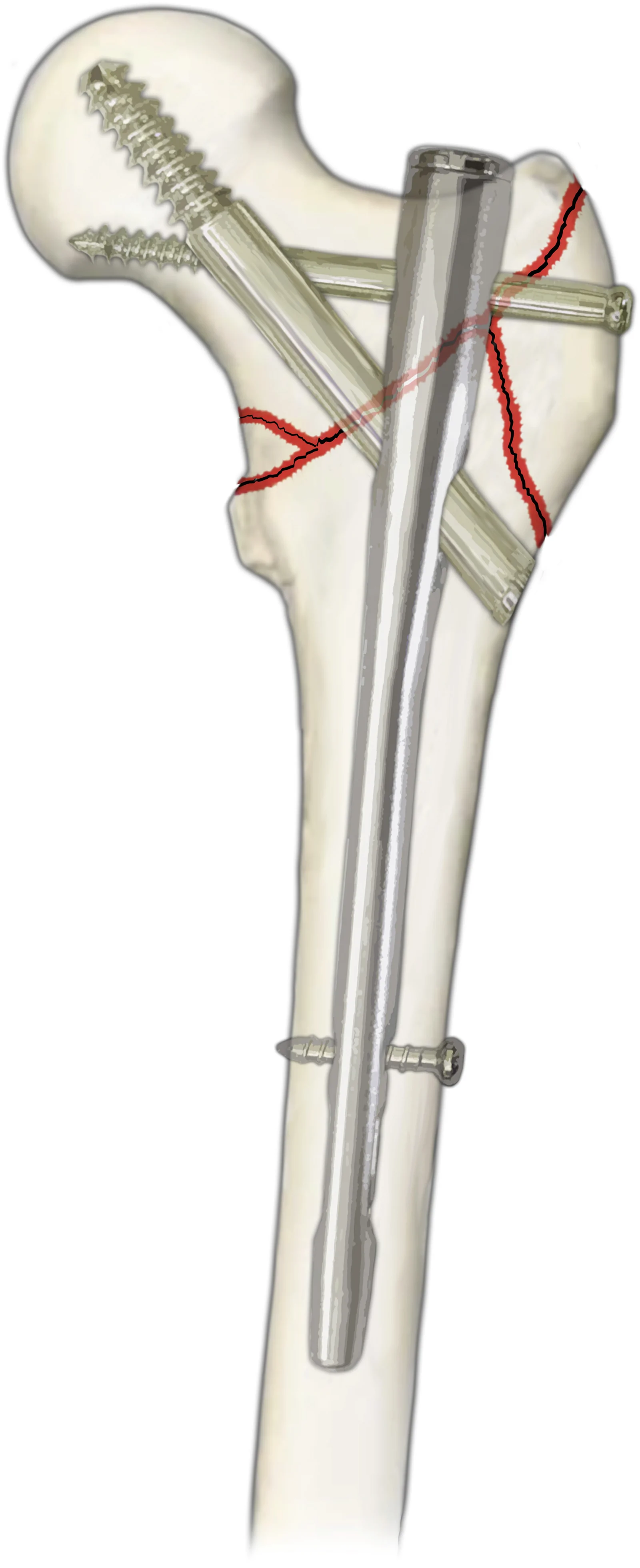
Illustration of Proximal Femoral Bionic Nail (PFBN).

Two commonly used intramedullary nails (IMN) for the treatment of IFFs are the Proximal Femoral Nail Antirotation (PFNA) and the InterTan nail. The PFNA is a single screw nail with a spiral blade that has a large surface area which increases the contact area with cancellous bone, thus providing stability and being favorable for patients with osteoporosis^7^^,^^8^ (Fig. 1). InterTan uses a dual screw integrated mechanism, which allows for increased rotational stability and resistance to varus collapse^8^^,^^9^ (Fig. 2). The Proximal Femoral Bionic Nail (PFBN) is a new IMN design that was introduced in the literature in the past couple of years, and the team that developed PFBN proposed a theory of “lever-reconstruction balance”. This theory suggests that the pressure trabeculae and tension trabeculae within the proximal femur form a shape that is similar to a lever, with the center of the femoral head being the fulcrum, and that this physiological lever is destroyed in a IFF.^10^ Based off this hypothesis, the PFBN remodels the lever and fulcrum using a triangular fixation system, which allows the proximal femur to be more effective in countering the compressive stress produced during weight-bearing, theoretically resulting in better healing^10^^,^^11^ (Fig. 3).

Although there are studies in the literature that compare the clinical outcomes and functional scores of different IMNs including PFNA and InterTan, the authors found no meta-analysis that compares PFBN against other IMNs. Therefore, the aim of this meta-analysis study is to compare the post-operative complications and functional scores between PFBN, PFNA, and InterTan in the treatment of IFF to see which IMN is most beneficial.

## 2 Methods

The preferred reporting items for systematic reviews and meta-analyses (PRISMA) guideline was used for this meta-analysis.^12^

### 2.1 Literature search

A literature search was carried out on PubMed and Embase up until April 2025. Due to the low search return in both databases in respect to PFBN studies, an additional search for PFBN comparison studies was carried out on Google Scholar. The following keywords were used to produce the preliminary search field: “intertrochanteric fracture”, “proximal femoral bionic nail” OR “PFBN”, “proximal femoral nail antirotation” OR “PFNA”, “InterTan nail”, “comparison”.

### 2.2 Inclusion and exclusion criteria

For a study to have been included in this meta-analysis, it must have met the following inclusion criteria: randomized controlled trial (RCT) or cohort study, compares at least two of the interested surgical interventions regarding IFF, reports at least one interested outcome. Outcomes of interest in this study included union time (weeks), weight-bearing time (days), hospital stay (days), operative time (minutes), intraoperative blood loss (mL), delayed union, non-union, hardware failures, superficial infection, deep infection, deep vein thrombosis (DVT), femoral head necrosis, screw cutout, Harris Hip Score (HHS) at 6 and 12-months post-operation. Studies that did not adhere to the criteria above and/or were case reports, meta-analyses, review papers, biomechanical studies, or did not have full texts available were excluded from this meta-analysis. Unfortunately, only three studies specifically compared PFBN to InterTan, resulting in a small sample size and omission of some outcomes of interest 13, 14, 15. To make up for this, the authors compiled included studies that had a PFBN group and those that had a InterTan group into a “Combined studies” group to carry out this specific comparison analysis, according to methods found in the Cochrane handbook.^16^

### 2.3 Assessment of study quality

The inclusion and exclusion criteria were independently applied to retrieved studies by each author. The Cochrane Risk of Bias Tool was used to assess the study quality for RCTs, which can be done with the Review Manager 5.4 software, and each study was scored as a low risk, unclear risk, or high risk of bias based on the following parameters: random sequence generation (selection bias), allocation concealment (selection bias), blinding of participants and personnel (performance bias), blinding of outcome assessment (detection bias), incomplete outcome data (attrition bias), selective reporting (reporting bias), and other bias.^17^ The Newcastle-Ottawa scale was utilized for all other studies.^18^

### 2.4 Data collection

Data collection included first author, publication year, journal, study type, treatment groups, sample sizes, number of stable and unstable fractures, and the outcomes of interest previously listed. According to the AO/OTA Fracture and Dislocation Classification, if a fracture was labeled as 31-A1, this was considered a stable fracture, while 31-A2 and 31-A3 were considered unstable. Two studies utilized the Evan's classification system, where there are five types of fractures.^14^^,^^15^ Types 1 and 2 were classified as stable fractures while types 3-5 were classified as unstable fractures.^19^^,^^20^ Nine studies were only available in Chinese as full texts ^14^^,^^15^^,^^21^, ^22^, ^23^, ^24^, ^25^, ^26^, ^27^. The authors initially utilized Google Translate as a screening tool for these studies, but each article was then verified by one of the authors who is a native speaker (JL).

### 2.5 Statistical analysis

Statistical analyses were carried out using Review Manager 5.4. Continuous variables were presented as mean ± standard deviation (SD), and dichotomous variables were presented as event rates. For one study, the SD was estimated from the interquartile range using an equation derived from the Cochrane handbook.^16^^,^^28^ Since there was a mixture of stable and unstable fractures in this study's sample population, the authors conducted a secondary analysis that only included unstable fractures in order to account for potential differences between the type of fractures. An inverse variance method with a mean difference (MD) was used for the continuous variables, while a Mantel-Haenszel method with risk ratio (RR) was used for the dichotomous variables. The I^2^ statistic was used to assess heterogeneity for each analysis with the following interpretation: 0% to 40% (might not be necessary), 30% to 60% (may represent moderate heterogeneity), 50% to 90% (may represent substantial heterogeneity), and 75% to 100% (considerable heterogeneity).^29^ If I^2^ ≤ 50%, a fixed effect analysis model was used. If I^2^ > 50%, a random effects analysis model was used. A P-value ≤0.05 was considered statistically significant, and significant results were presented as a forest plot with a 95% CI.

## 3 Results

### 3.1 Characteristics of included studies

**Fig. 4:**
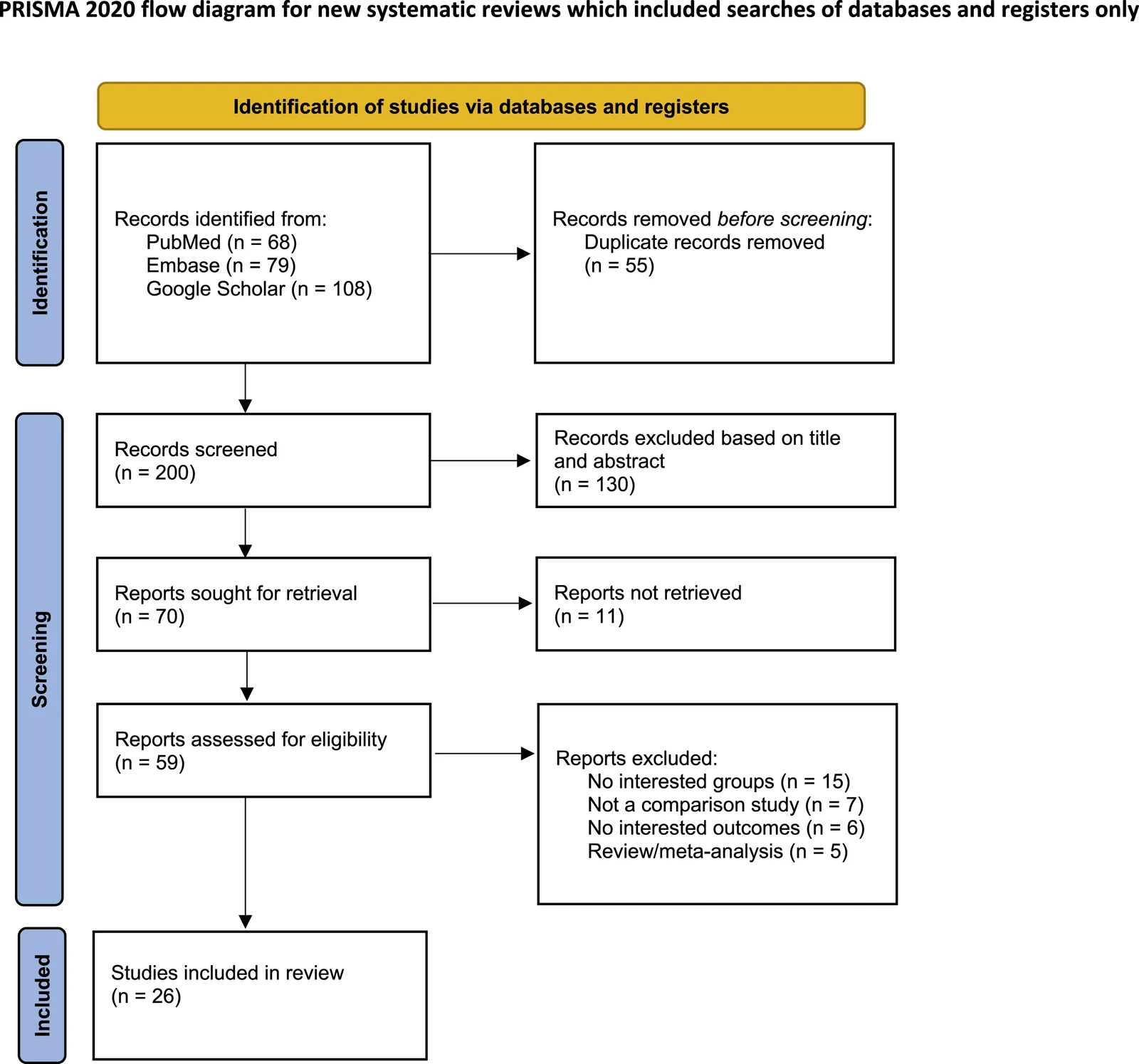
PRISMA (Preferred Reporting Items for Systematic Reviews and Meta-Analyses) flowchart.

A total of 26 studies were included in this meta-analysis (Fig. 4) 13, 14, 15^,^^21^, ^22^, ^23^, ^24^, ^25^, ^26^, ^27^, ^28^^,^^30^, ^31^, ^32^, ^33^, ^34^, ^35^, ^36^, ^37^, ^38^, ^39^, ^40^, ^41^, ^42^, ^43^, ^44^. There were 25 cohort studies and one RCT, with a total of 2690 patients, with 280 patients treated with PFBN, 1306 treated with PFNA, and 1104 treated with InterTan (Table 1). Two studies did not specify whether a patient had a stable or unstable fracture.^33^^,^^35^ Excluding these two studies, 300 patients had a stable fracture, and 2298 had an unstable fracture.

**Table 1:** Characteristics of included studies.

Author (Year)	Journal	Study type	Comparison	PFBN	PFNA	InterTan	Stable fractures	Unstable fractures
Fu (2024)^13^	J Musculoskelet Neuronal Interact	RCS	PFBN vs PFNA vs InterTan	22	40	20	33	49
Gavaskar (2018)^30^	J Orthop Trauma	RCS	PFNA vs InterTan	NA	50	50	0	100
Han (2024)^23^	Adv in Clin Med	RCS	PFBN vs PFNA	30	39	NA	0	69
Imerci (2018)^31^	Acta Orthop. Belg	RCS	PFNA vs InterTan	NA	33	36	0	69
Jia (2023)^14^	J of Clin Med in Pract	RCS	PFBN vs InterTan	25	NA	20	14	31
Jin (2024)^15^	Chin J Orthop Traumatol	RCS	PFBN vs PFNA vs InterTan	25	55	40	16	104
Kothiyal (2022)^32^	Int J Res Orthop	PCS	PFNA vs InterTan	NA	52	50	26	76
Kumar (2024)^33^	J Bone Joint Dis	PCS	PFNA vs InterTan	NA	25	27	NA	NA
Li (2022)^27^	Chin J Orthop Trauma	RCS	PFBN vs PFNA	46	46	NA	11	81
Li (2024)^34^	Am J Transl Res	RCS	PFNA vs InterTan	NA	26	26	10	42
Li (2024)^28^	Pak J Med Sci	RCS	PFNA vs InterTan	NA	78	73	0	151
Li (2025)^35^	Minerva Surg	PCS	PFNA vs InterTan	NA	20	20	NA	NA
Lin (2022)^21^	Chin J Trauma	RCS	PFBN vs PFNA	20	25	NA	10	35
Ling (2023)^22^	Chin J Orthop Trauma	RCS	PFBN vs PFNA	28	28	NA	6	50
Seyhan (2015)^36^	J Orthop Sci	RCT	PFNA vs InterTan	NA	43	32	18	57
Ülkü (2019)^37^	Bezmialem Science	RCS	PFNA vs InterTan	NA	16	12	0	28
Varmis (2025)^38^	PLoS One	RCS	PFNA vs InterTan	NA	76	118	52	142
Yang (2023)^24^	Chin J of Rep and Recon Surg	RCS	PFBN vs PFNA	24	24	NA	11	37
Yang (2024)^25^	J of Clin Med in Pract	RCS	PFBN vs PFNA	20	20	NA	14	26
Yu (2016)^39^	J Orthop Surg Res	RCS	PFNA vs InterTan	NA	72	75	0	147
Zehir (2015)^40^	Ulus Travma Acil Cerrahi Derg	RCS	PFNA vs InterTan	NA	85	88	0	173
Zhang (2017)^41^	J Int Med Research	RCS	PFNA vs InterTan	NA	88	86	79	95
Zhang (2017)^42^	J Orthop Surg Res	RCS	PFNA vs InterTan	NA	115	124	0	239
Zhang (2018)^44^	J Int Med Res	RCS	PFNA vs InterTan	NA	164	162	0	326
Zhang (2024)^26^	Chin J Orthop Trauma	RCS	PFBN vs PFNA	40	43	NA	0	83
Zhu (2023)^43^	Pak J Med Sci	RCS	PFNA vs InterTan	NA	43	45	0	88

RCS: Retrospective cohort study; PCS: Prospective cohort study; RCT: Randomized Controlled Trial; PFBN: Proximal Femoral Bionic Nail; PFNA: Proximal Femoral Nail Antirotation; NA: Not applicable.

### 3.2 PFBN versus PFNA

**Fig. 5:**
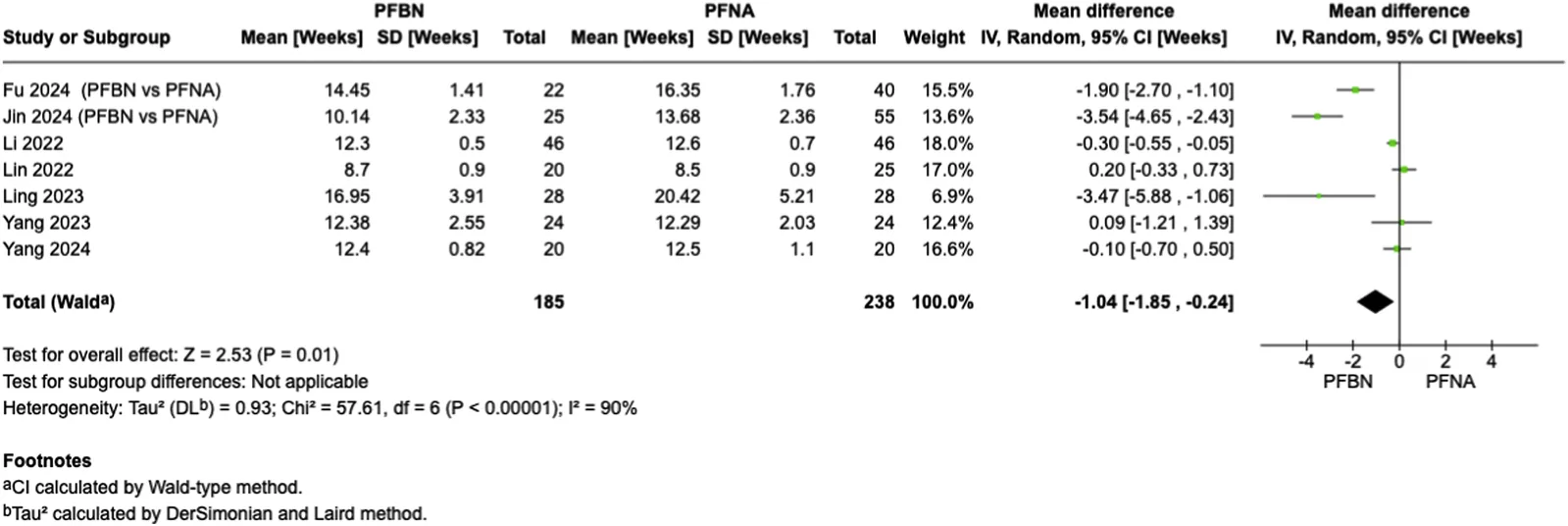

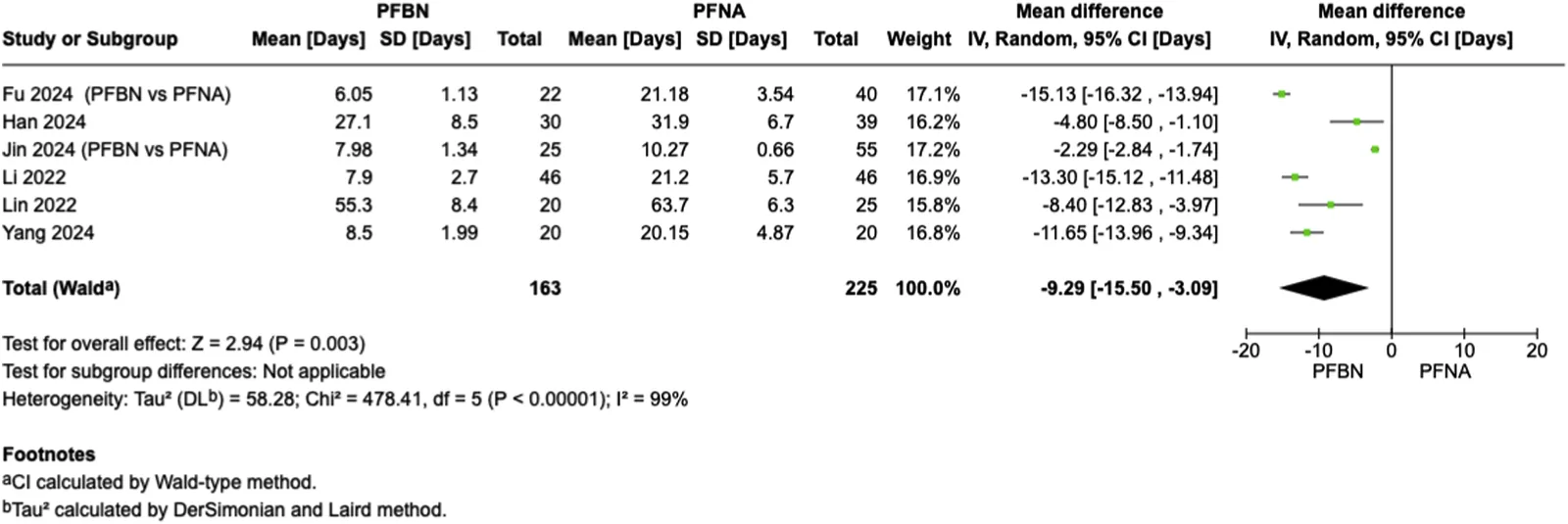

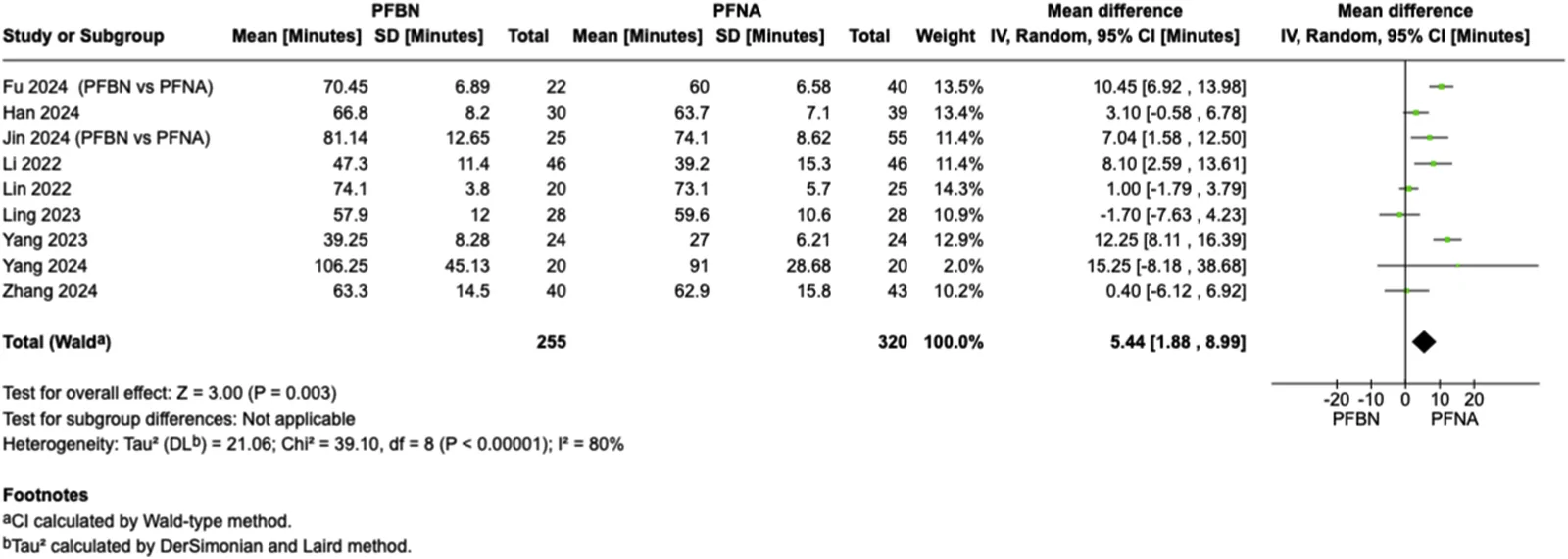

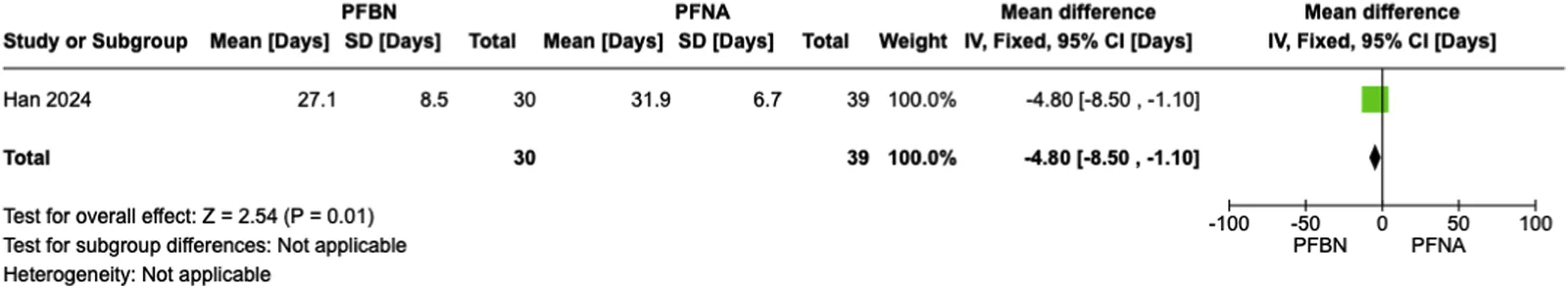

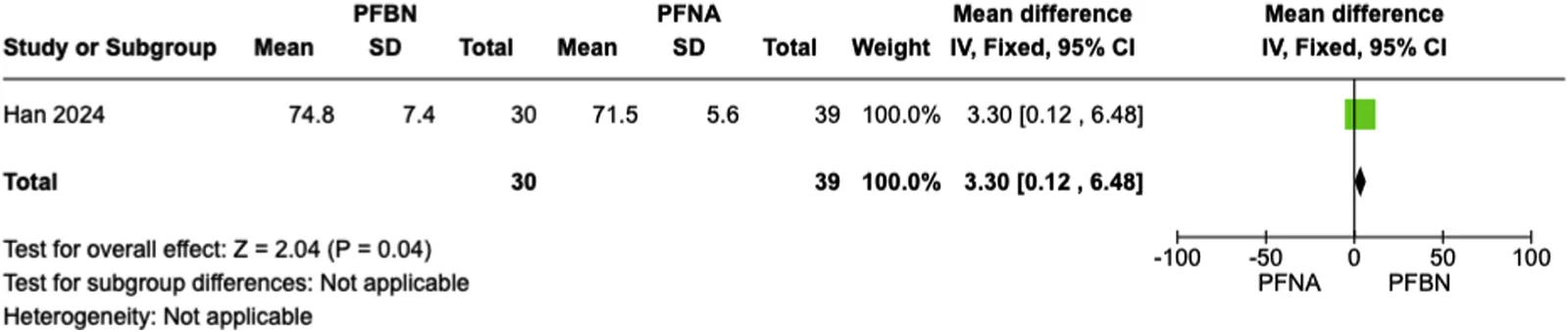
Forest plots for significant results in the comparison of PFBN versus PFNA, including (A) Union time, (B) Weight-bearing time, (C) Operative time, (D) Secondary analysis of weight-bearing time, (E) Secondary analysis of HHS 6-months post-operation.

When comparing PFBN to PFNA, significant differences were found in union time (MD = −1.04; 95% CI: −1.85 to −0.24; P = 0.01; Fig. 5A) and weight-bearing time (MD = −9.29; 95% CI: −15.50 to −3.09; P = 0.003; Fig. 5B) in favor of PFBN, while a significant difference was found in operative time (MD = 5.44; 95% CI: 1.88 to 8.99; P = 0.003; Fig. 5C) in favor of PFNA. No significant differences were found in the other outcomes.

In the secondary analysis, there were significant differences in weight-bearing time (MD = −4.80; 95% CI: −8.50 to −1.10; P = 0.01; Fig. 5D) and in HHS 6-months post-operation (MD = 3.30; 95% CI: 0.12 to 6.48; P = 0.04; Fig. 5E) in favor of PFBN. There were either no significant differences or data was unavailable in the other outcomes.

### 3.3 PFBN versus InterTan

When comparing PFBN to InterTan, significant differences were found in union time (MD = −2.62; 95% CI: −3.21 to −2.03; P < 0.001), weight-bearing time (MD = −9.58; 95% CI: −15.04 to −4.12; P < 0.001), hospital stay (MD = −2.37; 95% CI: −3.01 to −1.73; P < 0.001), operative time (MD = −6.85; 95% CI: −9.95 to −3.75; P < 0.001), intraoperative blood loss (MD = −52.43; 95% CI: −58.87 to −45.99; P < 0.001), HHS 6-months post-operation (MD = 3.11; 95% CI: 1.04 to 5.18; P = 0.003), and HHS 12-months post-operation (MD = 2.41; 95% CI: 0.60 to 4.22; P = 0.009) in favor of PFBN. No significant differences were found in the other outcomes.

In the secondary analysis, there were significant differences in hospital stay (MD = −2.08; 95% CI: −2.72 to −1.44; P < 0.001) and intraoperative blood loss (MD = −60.35; 95% CI: −71.62 to −49.08; P < 0.001) in favor of PFBN. There were either no significant differences or data was unavailable in the other outcomes.

### 3.4 PFNA versus InterTan

When comparing PFNA to InterTan, significant differences were found in union time (MD = 0.91; 95% CI: 0.37 to 1.45; P = 0.001), weight-bearing time (MD = 4.22; 95% CI: 0.32 to 8.12; P = 0.03) and screw cutout (RR = 4.81; 95% CI: 2.53 to 9.12; P < 0.001) in favor of InterTan, while significant differences were found in operative time (MD = −7.54; 95% CI: −12.40 to −2.68; P = 0.002) and intraoperative blood loss (MD = −15.80; 95% CI: −26.65 to −4.96; P = 0.004) in favor of PFNA. No significant differences were found in the other outcomes.

In the secondary analysis, there were significant differences in union time (MD = 0.91; 95% CI: 0.22 to 1.61; P = 0.01) and screw cutout (RR = 5.75; 95% CI: 2.67 to 12.39; P < 0.001) in favor of InterTan while there were significant differences in operative time (MD = −9.34; 95% CI: −16.29 to −2.40; P = 0.008) in favor of PFNA. There were either no significant differences or data was unavailable in the other outcomes.

## 4 Discussion

IFFs are debilitating injuries that are associated with significant morbidities and mortalities, especially in the elderly population. The PFBN is a new IMN that has recently been introduced and comparisons with other IMNs have not been thoroughly explored. Therefore, the purpose of this meta-analysis is to provide a detailed review on the differences in post-surgical results between PFBN, PFNA, and InterTan in the treatment of IFFs.

The authors were not able to find any meta-analysis studies that compared PFBN to PFNA in the literature. Thus, the results of this study provide new details on which IMN would be most beneficial for patients with IFFs. The authors believe that the most likely reason for the results seen in this study is due to the biomechanical differences between the two IMNs. The triangular nature of the PFBN should allow the proximal femur to augment stability and reduce stress at the fracture site, which could enable earlier weight-bearing and faster fracture healing.^11^ Regarding the operation time, the authors conclude that there are a couple of different reasons for this result. First, the PFBN has a more complex implant design compared to PFNA, which could potentially prolong the operative time. Second, the PFNA is a well-established IMN in comparison to PFBN. One could say that surgeons are more familiar with PFNA and thus are able to complete the procedure more effectively than with PFBN, where additional time might be necessary. This study's secondary analysis results were similar but also found a significant difference in HHS 6-months post-operation favoring PFBN. Better functionality is also most likely due to the triangular design of PFBN that provides additional support compared to PFNA.

Similarly, no meta-analyses were found that compared PFBN to InterTan in the treatment of IFFs. Regarding the outcomes of union time, weight-bearing time, and HHS at 6 and 12-months post-operation, the reasons for these differences are similar to the ones outlined above. When concerning average hospital stay, this is most likely correlated with the benefits of faster healing and enhanced stability that PFBN offers compared to InterTan, minimizing the demand for a prolonged hospital stay. Concerning the outcomes of operative time and intraoperative blood loss, these two are most likely related in that a shorter operation is often associated with lower intraoperative blood loss. InterTan could be associated with higher intraoperative blood loss due to its dual lag screw system which requires pinpoint positioning and possibly more surgical exposure, with one study reporting that InterTan was associated with higher amounts of perioperative hidden blood loss compared to other IMNs.^45^ The secondary analysis also found significant differences in hospital stay and intraoperative blood loss in favor of PFBN.

Regarding union time between PFNA and InterTan, the authors found one meta-analysis in the literature that favored InterTan, similar to this study's result.^46^ However, other studies have reported no significant differences in healing time between the two IMNs 47, 48, 49, 50. The dual screw integrated mechanism alongside the increased stiffness and bearing capacity of InterTan could be attributable to the faster union time compared to PFNA.^8^ However, healing time should be investigated further due to the differences seen in the literature. One meta-analysis reported no difference in weight-bearing time. Therefore, the authors look forward to additional studies being done that investigate this outcome.^49^ Three meta-analysis studies reported a significant difference in screw cutout in favor of InterTan, which aligns with the result from this study, although one study reported in favor of PFNA.^46^^,^^47^^,^^51^^,^^52^ InterTan likely has a lower screw cutout rate due to its biomechanical properties that allow for enhanced stability that lowers the risk of screw migration and cutout. When concerning average operative time, most meta-analysis studies found a significant difference in favor of PNFA, which was also seen in this study ^46^^,^^48^, ^49^, ^50^, ^51^, ^52^. Two studies found no significant difference.^47^^,^^53^ Concerning intraoperative blood loss, four meta-analysis studies found a significant difference in favor of PFNA.^48^^,^^49^^,^^51^^,^^53^ Three studies found no significant difference.^47^^,^^50^^,^^52^ The difference in operative time is likely due to the design and technique of each respective IMN. In comparison to the dual screw mechanism of InterTan, the PFNA has a simpler design with a single helical blade which possibly reduces the number of surgical steps required for insertion. Additionally, PFNA has been found to have a shorter fluoroscopy time compared to InterTan, which would also shorten the total operative time. It is probable that shorter operative times are correlated with lower intraoperative blood loss seen in PFNA as well, although this outcome should be explored further due to the discrepancy in the literature. This study found that the results from the primary and secondary analyses were mostly similar except the significant difference seen in HHS 6-months post-operation between PFBN and PFNA and the omission of some outcomes. This is most likely due to the fact that only a small portion of the primary analysis sample group were categorized as stable fractures, with the majority being unstable.

This study was not without its limitations. The main limitation the authors would like to point out is the small sample size of PFBN compared to the other IMNs. Due to PFBN being a recent innovation compared to the well-established implants like PFNA and InterTan, there are far lesser studies that include PFBN as a comparison group. This study also included both stable and unstable fractures, which could potentially introduce bias due to possible differences in treatment outcomes based on the stability of the fracture. However, the authors believe that since the number of stable fractures were a small percentage of the total sample size, the bias would be minimal. And to account for this, a secondary analysis was carried out that only included unstable fractures, yielding similar results. Furthermore, due to the combining of groups for the PFBN versus InterTan analysis, this study was not able to report heterogeneity for those analyses. Although this could lead to restricted generalizability, the authors hope that additional studies are carried out in the future to further investigate this comparison. Finally, the majority of included studies were cohort studies, with only one study being an RCT. Due to RCTs having better methodological designs that account for bias and confounding, the lack of in this study could potentially introduce bias and impact the validity of the results.

## 5 Conclusion

PFBN showed superior outcomes in fracture healing and weight-bearing time compared to PFNA and InterTan, while also presenting with shorter hospital stay, shorter operative time, lower intraoperative blood loss, and better functional scores compared to InterTan, proving to be a viable new alternative for intramedullary fixation in patients with IFFs. InterTan revealed preferable post-operative outcomes compared to PFNA, wish a faster time to fracture healing and weight-bearing and lower incidence of cutout, while PFNA had shorter operative times and lower intraoperative blood loss. Overall, this meta-analysis found PFBN to be the preferred treatment method, with both PFNA and InterTan proving to be effective alternatives. Additional studies that compare PFBN to other IMNs that have substantial sample sizes and finer methodological design should be carried out to further investigate the differences in outcomes and functional scores between different methods IMNs for the treatment of IFFs.

## Declaration of patient consent form

Not applicable.

## Financial support and sponsorship

The authors declare no competing financial interests.

## Ethical statement

We confirm that all authors have read and approved the manuscript, and no other individuals meet the authorship criteria but are not listed. We have also agreed upon the order of authorship as presented. Furthermore, we assure you that this manuscript has not been submitted elsewhere.

Thank you for considering our work for publication. We deeply appreciate the time and effort you and the journal's esteemed review team will invest in our manuscript. Please feel free to reach out if you have any questions or require additional information.

## Guardian patient's consent

NA.

## Credit author statement

JL & DY contributed to the conception and design of the study. TC, KD, and NJ performed the literature search and data extraction. TC, KD, NJ, DY & JL conducted the data analysis, drafted the initial manuscript, and revised the manuscript. JL & DY critically revised the manuscript for important intellectual content. JL supervised the study, provided guidance throughout, and finalized the manuscript. All authors read and approved the final version of the manuscript and agree to be accountable for all aspects of the work.

## Funding information

This research was conducted independently and did not receive specific funding from public, commercial, or not-for-profit agencies.

## Disclosure of potential conflicts of interest

Thomas Cho, Kriti Devgan, and Jiayong Liu declare that they have no conflict of interest.
